# The Changing Landscape in Upper Limb Sports Rehabilitation and Injury Prevention

**DOI:** 10.3390/sports11040080

**Published:** 2023-04-07

**Authors:** Eleftherios Paraskevopoulos, George M. Pamboris, Maria Papandreou

**Affiliations:** 1 Department of Physiotherapy, University of West Attica, 12243 Athens, Greece; 2 Laboratory of Advanced Physiotherapy, University of West Attica, 12243 Athens, Greece; 3 Department of Health Sciences, School of Sciences, European University Cyprus, Nicosia 2404, Cyprus

## 1. Editorial

This editorial aims to feature authors who intend to submit their research to this Special Issue of *Sports* entitled “Clinical Advances in Upper Limb Sports Rehabilitation and Injury Prevention” in areas that need special consideration. This Special Issue may attract investigators that are implementing research in this area. Research papers that examine the risk of injury assessment strategies, injury prevention or rehabilitation programmes in overhead athletes are also welcome. This editorial aims to assist authors in considering several factors when designing and submitting their research to increase the methodological quality and adequately support their findings.

## 2. Injury Prevention and Assessment

In overhead sports, high loads and forces are created during ball serving or hitting in positions at the extreme range of motion, which may lead to pathological changes within the shoulder joint structure and high irritability [1]. In recent years, more and more sports physiotherapists have incorporated several tests, assessment techniques and prevention exercise programmes for overhead athletes at high risk of injury [2]. Experts agree that prevention strategies, rehabilitation and return to sports programmes are not separate fields and are highly interrelated [2]. However, these fields tend to be studied separately in sports injury research, creating confusion among clinicians willing to apply new knowledge in their clinical practice. For example, in the case of assessing an overhead athlete for injury risk, identifying the risk is not sufficient without guiding the athlete on ways to reduce the identifiable risk.

Furthermore, a risk of injury assessment is usually based on qualitative assessment through the execution of several exercises with a pass or fail component, such as the Functional Movement Screen (FMS) tool [3], or by assessing psychosocial factors that increase the risk of injury, such as Kinesiophobia, through questionnaires (i.e., the Tampa Scale for Kinesiophobia), or through a quantitative assessment based on several cutoff values for the range of motion (ROM) or muscle strength ratios [4]. Researchers should rely on a specific range of values for the latter and avoid using different values in each research study. For example, cutoff values for ROM, strength and on-field testing performance have recently been provided [2]. These include normal values and the minimum detectable change (MDC) for shoulder internal rotation (IR) and external rotation (ER), total shoulder ROM, rotator cuff strength, including internal and external rotators as well as ER/IR strength ratios. Furthermore, normal minimum values and MDC for functional tests, including the Seated Medicine Ball Throw, the Upper Quadrant Y-balance test and the Closed Kinetic Chain Upper Extremity Stability test, have been reported [2]. All of these tests have shown to be reliable, easy and quick to administer and can be good alternatives to more expensive isokinetic testing [5,6]. These tests should be used as a reference in studies evaluating shoulder-related tests’ diagnostic properties and assessment procedures.

The above objective measures and procedures may be more valuable if they are combined with Patient-Reported Outcome Measures (PROMs). Studies have shown that despite the important clinical utility of PROMs in sports medicine, only 15% to 26% of sports therapists and athletic trainers routinely use these instruments to assess their athletes [7]. Research has shown that PROMs designed for overhead athletes, such as the Kerlan–Jobe Orthopaedic Clinic overhead athlete score and the Functional Arm Scale for Throwers, are reliable, valid, and responsive in this population [8,9]. Furthermore, not only PROMs designed for overhead athletes but several PROMs designed for more general populations, such as the Disabilities of the Arm, Shoulder and Hand Questionnaire, the Quick Disabilities of the Arm, Shoulder and Hand Questionnaire and the Upper Extremity Functional Instrument are also appropriate, acceptable, and feasible for use in sports clinical settings [7]. The authors of this editorial encourage researchers to use PROMs in future studies.

Another important aspect of screening in sports injury prevention programmes is related to the conditions in which each screening tool is evaluated. For example, based on previous research, tools such as the FMS have limited ecological validity since reliability is examined by studies that are not designed to simulate the conditions in which these assessment procedures might normally be conducted in routine practice [10]. Thus, researchers should think about examining the ecological validity of any of the tools used for injury prevention in this population of athletes. Of course, when considering aspects that may improve the ecological validity of any screening tool, researchers should examine the methodological characteristics of each study in order to resemble conditions encountered in sports clinical settings.

Some injury prevention programmes have also been investigated in terms of effectiveness by a few authors [5,6,7,8,9]. Some of these programmes include the Oslo Sports Trauma Research Center (OSTRC) Shoulder Injury Prevention Programme for handball players [11], the Advanced Throwers Ten Exercise Program for overhead throwing athletes [12] and the FIFA 11+ shoulder injury prevention program for goalkeepers [13] and volleyball players [14,15]. The above programmes have shown a statistically significant reduction in injury occurrence compared to control groups. However, the methodological shortcomings of these studies may not allow the application of these prevention programmes in clinical practice and highlight the need for further investigation. The limitations of the aforementioned studies include the recruitment of amateur athletes only, thus limiting generalizability in professional athletes [13], a non-standardized exercise programme in the control groups, indicating possible within-group variation [7], the inclusion of injured players in the study at baseline and a between-group variation in the risk of injury (i.e., higher risk of injury of the athletes in the control group) [11] and the identification of efficacy based on anecdotal evidence only [12]. These limitations should be considered in future studies evaluating the effectiveness of injury prevention programmes.

As for the implementation and structure of future studies, researchers are advised to use the cycle of injury prevention when evaluating the effectiveness of an injury prevention programme [2,16]. This cycle includes four steps that start with (1) problem identification, including epidemiological data and rates of injury occurrence in a specific athletic population, continues (2) with a report on the risk factors and injury mechanisms, including modifiable (strength, balance, joint mobility and biomechanics, etc.) and non-modifiable (age, gender, etc.) risk factors, (3) the examination of an injury prevention exercise programme or strategy that should be examined prospectively in similar groups of athletes and by taking into consideration the limitations of the studies mentioned above, and the last step, (4) a repetition of step 1 to evaluate whether the prevention programme resulted in better outcomes in terms of injury rates. Researchers should also consider factors such as adherence to the injury prevention programme and altering the modifiable risk factors when implementing the injury prevention programme under study. Additionally, from a clinical perspective, assessing who can supervise and implement the programme (i.e., the coach, the physiotherapist, or if it can be performed without supervision).

## 3. Rehabilitation

The rehabilitation of an overhead athlete should include a thorough assessment in clinical practice. However, this should also be the case in research studies that aim to examine the effectiveness of an exercise programme to ensure the homogeneity of the groups under comparison. This includes the pathological structures involved, irritability, stage of pathology and the individual characteristics of the athletes (i.e., the level of the athlete, the frequency of exposure to the sport, position, training programme phase, etc.). Additionally, exercise programmes in either group (intervention or control) should be designed and reported based on the FITT (frequency, intensity, time and type) principle [17,18]. This will allow a better understanding of the parameters of the interventions and will also allow replication in future studies and clinical practice.

Several studies have examined the effectiveness of different exercise programmes for injured overhead athletes. These generally include a four-phase rehabilitation programme consisting of the acute phase, where the clinician aims to promote analgesia, reduce inflammation, increase ROM and rotator cuff strength and improve tissue flexibility. In the intermediate phase, the clinician seeks to achieve full ROM and target the kinetic chain. However, the advanced strengthening phase aims to improve strength and endurance and retrain throwing biomechanics. In the final rehabilitation phase, the return to activity phase, the goal is to ensure that the athlete can safely return to their sporting activity by using throwing exercises and flexibility drills that imitate the intended sport [19].

Although there are several proposed protocols for the management of an injured overhead athlete and by considering the multidimensional nature of each relevant sport, controversy still exists concerning several interventions that have been used in the past. For example, stretching for improving glenohumeral IR deficit (GIRD) has been questioned in previous research studies [2,20], suggesting that instead of stretching for improving GIRD, clinicians should try to strengthen the external rotators, which are the decelerator mechanism during throwing activities, especially the infraspinatus muscle [21]. This question has not been adequately answered yet through research, and researchers should aim to investigate this hypothesis.

Furthermore, addressing the kinetic chain has recently been encouraged recently regarding overhead professional athletes [4,22,23,24]. However, there is still some controversy regarding the possible additional benefits of the kinetic chain approach over the more traditional exercises used in shoulder rehabilitation. Adding kinetic chain exercises into the exercise programme seems to improve axioscapular muscle recruitment, produce lower trapezius muscle ratios and reduce the demands on the rotator cuff muscles [25]. Nevertheless, studies on the effectiveness of kinetic chain approach exercises remain scarce; thus, more studies are needed.

Motor control/retraining exercises have also been recommended in the past [26] and in a recent consensus [27]. However, less is known about the effectiveness of various types of motor learning on motor control. Several components of motor learning, such as Amount of Practice, Whole vs. Part Practice, Constant vs. Variable Practice, Mental Practice, Specificity and Location of Practice and the role of Feedback, including Composition of Feedback Mode of Delivery, Timing of Feedback, Frequency of Feedback and Precision of Feedback have not been investigated in the injured overhead athlete [28].

Researchers should examine the effectiveness of each intervention not only based on clinical outcomes related to the symptomatology of the injured athletes but also on their ability to return to play. Considering that short-term rehabilitation may result in re-injury and that long-term rehabilitation is sometimes unrealistic for a professional athlete, investigating the effectiveness of interventions on factors related to return to play is extremely important. This can be assessed either through specially designed on-field tests or/and through the assessment of Psychological Components of Athlete Readiness [27].

## 4. Summary

Researchers designing experimental studies to investigate techniques for assessing and managing the overhead injured athlete are encouraged to use the suggestions in this editorial to enhance the quality of their research, allowing them to draw safe conclusions for implementation in clinical practice. Innovative sports performance analyses that incorporate new technologies (artificial intelligence, VR 3D motor control or cognitive techniques, etc.) are also welcomed in this Special Issue.
